# Supplementary material for: Comparative genomic profiling of SLC26A4-expressing cells in the inner ear and other organs
Source: PLoS One. 2025 Feb 11;20(2):e0318972. doi: 10.1371/journal.pone.0318972 (PMC11813142; doi:10.1371/journal.pone.0318972)
Supplement: Attachment — Submitted filename: Response_to_Reviewers.docx [file pone.0318972.s001.docx]

**Response to Reviewers**
Manuscript Title: Comparative genomic profiling of SLC26A4-expressing cells in the inner ear and other organs
Manuscript ID: PONE-D-24-55010

**Dear Dr. Nejat Mahdieh and Reviewers,**

We are grateful for the opportunity to revise and resubmit our manuscript. We sincerely thank you for your constructive and insightful comments, which have greatly helped us improve the clarity and scientific rigor of the manuscript. Below, we provide a point-by-point response to each comment raised by the reviewers and the editorial office. For ease of reference, we have included the reviewers’ comments in bold, followed by our detailed responses in plain text.

We have also updated the manuscript to reflect these changes, with all revisions clearly marked in the "Revised Manuscript with Track Changes" file. A clean version of the revised manuscript has also been provided for your convenience.

We believe these revisions have significantly enhanced the manuscript and hope it meets the high standards of PLOS ONE. Please let us know if further clarification or additional modifications are required.

Thank you again for your thoughtful feedback and guidance throughout the review process.

Sincerely,
Keiji Honda
On behalf of all authors

**Reviewer #1**

**This article examines the association between Pendred syndrome and autosomal recessive nonsyndromic hearing loss type 4 (DFNB4) with SLC26A4 gene mutations. The aim of this study was to evaluate the gene profiles of Slc26a4-expressing cells in mice to better understand the regulatory mechanisms and functions of this gene.
The following suggestions are made for improvement:**

1. **Abstract: It is recommended to highlight the key findings in the abstract and to state them more precisely.**

Thank you for your suggestion. We have revised the abstract to emphasize the key findings more explicitly, focusing on the novel insights provided by our study, while ensuring it remains within the 300-word limit.

2. **Introduction: At the end of the introduction, it is recommended to explain what new information this research may contribute to our current knowledge.**

We have revised the last paragraph at the end of the Introduction section outlining the novel contributions of our study, particularly how it advances the understanding of the role of SLC26A4 in various tissues and the development of novel therapeutic strategies for Pendred syndrome/DFNB4 or other SLC26A4 related diseases.

3. **Discussion: The discussion is the most important part of an article, so please consider the following to improve it:**

- **Although some relevant sources are included in the discussion, referencing existing past research can enhance the quality of the discussion. It is recommended to compare the results of your study with those of other studies and to present hypotheses regarding the consistency or inconsistency of the evaluation results.**

Thank you for your constructive feedback. In response, we have added a new paragraph to the Discussion section that references previous studies on proteins interacting with SLC26A4, specifically IQGAP1 and the μ2 subunit of the AP-2 complex. These proteins have been reported to influence SLC26A4 trafficking and functionality through distinct mechanisms, as demonstrated in earlier yeast two-hybrid and pull-down assays. By incorporating data from the current study, we highlight the ubiquitous expression patterns of *Iqgap1* and *Ap2m1* and propose that their roles in modulating SLC26A4 are likely context-dependent, influenced by cell-specific signaling pathways and cofactors. This addition provides a comparative perspective that underscores the complexity of SLC26A4 regulation and aligns with your recommendation to enhance the discussion with relevant past research and hypotheses.　We believe this addition strengthens the Discussion section and addresses your comment effectively.

- **Finally, while the limitations of this study are mentioned, which is very good, provide suggestions for future studies to other researchers.**

we have added a new paragraph to the Discussion section that outlines two potential directions for future research. These include pooling *Slc26a4*-expressing cells for bulk RNA-seq analysis to better understand their transcriptomic landscape and utilizing more sensitive single-cell RNA-seq methods to detect low-abundance transcripts and explore cellular heterogeneity. We hope this addition provides valuable guidance for future studies.

**Reviewer #2**

**The introduction addresses an important genetic factor related to hearing loss and associated syndromes, which is relevant to both clinical and research communities. The introduction sets the stage for understanding the significance of investigating SLC26A4 in different cell types and organs. However, there are some areas to improvement: Additional details on the mechanisms by which SLC26A4 dysfunction leads to hearing loss could enhance the reader's understanding of its importance. In addition, including prevalence statistics for Pendred syndrome and DFNB4 would strengthen the case for why this research is significant and necessary.　In conclusion, while it is a suitable introduction, refining it based on the points mentioned would significantly enhance its quality and impact.**

We sincerely thank the reviewer for their thoughtful and constructive feedback on the Introduction section. We have expanded the Introduction to include a detailed explanation of the biological mechanisms linking SLC26A4 dysfunction to hearing loss, referencing a 2011 review that a foundational hypothesis for these mechanisms. We also have incorporated prevalence data for Pendred syndrome and DFNB4 to emphasize the clinical significance and relevance of our study.

**Regarding methods, it is undeniable fact that all parts are acceptable and ethics statement is clear and concise, appropriately indicating that no live animals were involved in the study. This transparency is commendable. however, there are some constrictive notes:**

- **The explanation of the elbow method for determining the number of principal components is appropriate, but including a brief discussion on how this method works could enhance clarity for readers less familiar with PCA. The use of the Louvain algorithm for community detection is a solid choice, and specifying the range for the resolution parameter adds valuable detail.**

We have added a brief explanation of how the elbow method works in the Methods section to improve clarity for readers less familiar with PCA.

- **The integration of datasets to minimize batch effects is well-described, highlighting the use of anchor detection and harmonization methods. However, it may be beneficial to discuss any potential limitations or assumptions made during this integration process, as batch effects can sometimes persist despite these efforts.**

In the Discussion section, we have expanded the limitations paragraph to address potential limitations and assumptions related to the integration process and batch effects. Specifically, we discuss how residual variability may persist despite anchor-based harmonization and highlight the need for standardized conditions in future studies to further minimize these effects.

**The methods and results sections are generally well-structured and informative. They provide a comprehensive overview of the analytical approaches used in this study while maintaining clarity. Suggestions for improvement include providing additional context or rationale for certain methodological choices, discussing potential limitations, and elaborating on key findings' implications. Overall, this article contributes valuable insights into the gene expression landscape of Slc26a4-expressing cells in the inner ear.**

Thank you for your positive and encouraging feedback on the methods and results sections. We appreciate your thoughtful suggestions, which have guided our revisions and improved the overall quality of the manuscript.

**Editorial Office Comments**

**1. Please ensure that your manuscript meets PLOS ONE's style requirements, including those for file naming. The PLOS ONE style templates can be found at 
https://journals.plos.org/plosone/s/file?id=wjVg/PLOSOne_formatting_sample_main_body.pdf and 
https://journals.plos.org/plosone/s/file?id=ba62/PLOSOne_formatting_sample_title_authors_affiliations.pdf**

Thank you for the reminder regarding PLOS ONE's style requirements. We have updated the manuscript file name and confirmed that the manuscript adheres to the journal’s style guidelines. Additionally, all figure file names have been updated, and the figures have been converted to TIFF format. We also adjusted the size and font size within the figures to ensure they meet the journal’s specifications.

**2. Please note that PLOS ONE has specific guidelines on code sharing for submissions in which author-generated code underpins the findings in the manuscript. In these cases, we expect all author-generated code to be made available without restrictions upon publication of the work. Please review our guidelines at https://journals.plos.org/plosone/s/materials-and-software-sharing#loc-sharing-code and ensure that your code is shared in a way that follows best practice and facilitates reproducibility and reuse.**

Thank you for highlighting the importance of code sharing to support reproducibility and reuse. In response, we have uploaded the R scripts used for data analysis in this study, including preprocessing, clustering, and visualization, to Figshare. The code is publicly available at Figshare with the DOI: 10.6084/m9.figshare.28190282. Additionally, we have added the following statement to the data availability statement:

*“The R scripts used for data analysis in this study, including preprocessing, clustering, and visualization, are publicly available at Figshare (DOI: 10.6084/m9.figshare.28190282).”*

**3. Thank you for stating the following in the Acknowledgments Section of your manuscript: This work was supported by JSPS KAKENHI Grant Number JP21K09602. We sincerely thank Yoshiyuki Kawashima and Taku Ito for their invaluable feedback and constructive review of this manuscript. We would like to thank Editage (www.editage.jp) for English language editing.
We note that you have provided funding information that is not currently declared in your Funding Statement. However, funding information should not appear in the Acknowledgments section or other areas of your manuscript. We will only publish funding information present in the Funding Statement section of the online submission form. 
Please remove any funding-related text from the manuscript and let us know how you would like to update your Funding Statement. Currently, your Funding Statement reads as follows: KH received funding from the Japan Society for the Promotion of Science (JSPS) KAKENHI Grant Number JP21K09602 (https://www.jsps.go.jp/english/). The funder had no role in study design, data collection and analysis, decision to publish, or preparation of the manuscript.**

**Please include your amended statements within your cover letter; we will change the online submission form on your behalf.**

We have removed the funding information from the Acknowledgments section and ensured it is only included in the Funding Statement section. The funding statement does not need to be changed.

**4. We note that you have included the phrase “data not shown” in your manuscript. Unfortunately, this does not meet our data sharing requirements. PLOS does not permit references to inaccessible data. We require that authors provide all relevant data within the paper, Supporting Information files, or in an acceptable, public repository. Please add a citation to support this phrase or upload the data that corresponds with these findings to a stable repository (such as Figshare or Dryad) and provide and URLs, DOIs, or accession numbers that may be used to access these data. Or, if the data are not a core part of the research being presented in your study, we ask that you remove the phrase that refers to these data.**

We have removed the phrase “data not shown” in the manuscript.
